# Tick-Borne Rhabdomyolysis: A Rare Case of Rhabdomyolysis and Acute Kidney Injury Due to Anaplasmosis

**DOI:** 10.7759/cureus.34835

**Published:** 2023-02-10

**Authors:** Megha Dogra, Manish Thakur, Amrat Kumar, Garima Thakur

**Affiliations:** 1 Internal Medicine, Mary Imogene Bassett Hospital, Cooperstown, USA; 2 Internal Medicine, Cayuga Medical Center, Ithaca, USA; 3 Internal Medicine, Indira Gandhi Medical College, Shimla, IND

**Keywords:** tick borne infections, infectious disease pathology, infectious disease, aki, anaplasmosis

## Abstract

Anaplasmosis is a tick-borne illness commonly seen in the northeastern states of the United States. The most common presenting signs are fever, malaise, and body aches accompanied by leukopenia, thrombocytopenia, and transaminitis. Rhabdomyolysis and acute kidney injury are rare presentations that can lead to significant morbidity.

We present the case of a patient who presented with non-specific symptoms of malaise, fatigue, and body aches and was found to have rhabdomyolysis and acute kidney injury on laboratory workup. A presumptive diagnosis of anaplasmosis was made, and the patient was started on treatment for the same. The patient recovered successfully.

Our case highlights the rare presentation of anaplasmosis with rhabdomyolysis and acute kidney injury. Physician awareness is needed for early diagnosis and preventing morbidity.

## Introduction

Anaplasmosis is a tick-borne disease caused by the bacterium Anaplasma phagocytophilum. This disease was previously known as human granulocytic ehrlichiosis (HGE). Constitutional symptoms, leukopenia, thrombocytopenia, and elevated liver function tests are known to be the typical presentations [1]. Rhabdomyolysis and acute kidney injury have been rarely reported as the initial presenting complaints. 

## Case presentation

A 69-year-old male presented to the hospital in October with malaise, fatigue, dizziness, and body aches. He did not have any known chronic medical conditions, was not taking any chronic medications, and was a resident of upstate New York. 

On arrival, vitals were: blood pressure of 104/84 mm Hg, a pulse of 98 beats per minute, a temperature of 36.2°C (97.1 °F), respiratory rate of 20 per minute, and SpO2 of 95% on room air. There was no rash or eschar. The following abnormalities were discovered during the lab workup: high-sensitivity Troponin I of 141.4 pg/ml (ref range = 20 pg/ml), creatine phosphokinase (CPK) of 24,078 U/L (122-218 U/L), creatinine (Cr) of 1.3 (baseline Cr of 0.8), blood urea nitrogen (BUN) of 30 mg/dl (7-25 mg/dl), bicarbonate of 19 mmol/L (21-31 mmol/L). 

The patient did not have any complaints of chest pain. No history of known coronary artery disease. The electrocardiogram showed a normal sinus rhythm with no changes to suggest ischemia. An echocardiogram showed a left ventricular ejection fraction of 55% and no wall motion abnormalities. The patient denied having any recent falls, new medication use, or seizure episodes. He was started on intravenous fluids. 

Lab workup on the second day of admission showed worsening leukopenia (1.5 x 10E3 cells/uL), thrombocytopenia (25 x 10E3 cells/uL), and Transamnitis (Aspartate aminotransferase/alanine aminotransferase: 442/100, alkaline phosphatase: 61, total bilirubin: 1.3). Given the high prevalence of tick-borne illnesses in the geographic location, polymerase chain reaction (PCR) for tick-borne diseases was obtained. While waiting for the PCR results, the patient was started on Doxycycline 100 mg twice daily. The following day, the patient reported improvement in his symptoms, which was accompanied by an improvement in his lab abnormalities. Table 1 shows laboratory workup on day 1, day 2, and day 4. 

**Table 1:** Laboratory workup on days 1, 2, and 4 CPK: Creatine phosphokinase, CO2: Bicarbonate, BUN: Blood urea nitrogen, ALT: Alanine transaminase, AST: Aspartate aminotransferase, MCV: Mean corpuscular volume, MCH: Mean corpuscular hemoglobin, MCHC: Mean corpuscular hemoglobin concentration

Investigations	Day 1	Day 2	Day 4	Reference Ranges	Unit
Troponin I	141.4	138.2	120	<= 20.0	pg/ml
CPK	24,078	19,515	997	122-218	U/L
Sodium	132	136	139	136-145	mmol/l
Potassium	4	3.8	3.6	3.5-5.1	mmol/l
Chloride	102	109	113	98-110	mmol/l
Glucose	109	105	83	70-139	mg/dl
CO2	19	20	19	21-31	mmol/l
Anion gap	11	7	7	6-14	mmol/l
BUN	30	27	24	7-25	mg/dl
Creatinine	1.3	0.9	0.8	0.7-1.3	mg/dl
ALT	321	442	383	7-72	U/L
AST	67	100	90	13-39	U/L
Alkaline phosphatase	53	61	54	34-104	U/L
Total bilirubin	0.8	1.3	0.9	0.3-1.0	mg/dl
white cell count	4.9	1.5	10.2	3.7-10.6	x 10E3 cells/uL
hemoglobin	14.2	13.2	13	11.5-18.0	g/dl
hematocrit	42.2	38.9	36.8	35.0-50.0	%
MCV	89.8	89.6	86.8	81.0-99.0	fL
MCH	30.2	30.4	30.3	27-33.5	pg
MCHC	33.6	33.9	34.8	31.5-35.5	g/dl
platelet count	47	25	39	140-425	x 10E3 cells/uL

Serial troponins were 138.2, 120 pg/ml, CPK improved with hydration, and Creatinine improved to 0.8. On day four of admission, PCR resulted positive for *anaplasmosis phagocytophilum*, which confirmed the suspicion of anaplasmosis, as shown in Table 2. 

**Table 2:** Tick-borne panel polymerase chain reaction result

Component	Result
Babesia microti	negative
Babesia duncani	negative
Babesia divergens/MO-1	negative
*Anaplasma *phagocytophilum	positive
Ehrlichia chaffeensis	negative
*Ehrlichia ewingii*/*canis*	negative
Ehrlichia muris eauclairensis	negative
*Borrelia miyamotoi* PCR, B	negative

The patient was discharged with instructions to complete 10 days of doxycycline.

After two weeks, the patient was evaluated in the clinic and had a complete resolution of symptoms, as well as normalization of lab abnormalities. 

## Discussion

Anaplasmosis is a systemic, tick-borne illness. In the eastern United States, the black-legged tick (*Ixodes scapularis*) and the western coast black-legged tick (*Ixodes pacificus*) are the primary vectors. *Ixodes scapularis* is also known to transmit other pathogens (e.g., *Borrelia burgdorferi*), and co-infections have been reported. White-tailed deer and white-footed mice are the most common reservoirs. Less commonly, there have been cases of disease transmission by blood transfusion [2] as well as organ transplant [3]. 

**Figure 1:**
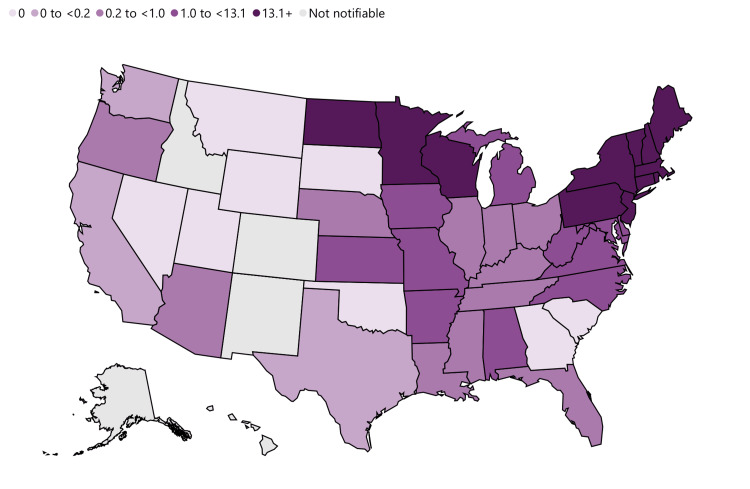
Annual incidence (per million population) of reported Anaplasmosis in United States Adapted from the Centers for Disease Control and Prevention website [4].

Anaplasmosis was first recognized as a human disease in the mid-1990s and became nationally notifiable by 1999. The number of cases reported to the centers for disease control and prevention (CDC) annually has been steadily increasing since the disease became reportable. There were 348 reported cases in 2000, followed by 5,762 in 2017 and 5,655 in 2019 [4]. In the United States of America (USA), the highest incidence has been reported in the northeastern states, including Connecticut, New York, Rhode Island, and Wisconsin [5]. Figure 1 shows the annual incidence of anaplasmosis per million population reported in 2019.

This geographic location corresponds to the prevalence of ticks. The peak seasons of transmission coincide with seasonal variations due to an increase in arthropod vectors and vertebrate host reservoirs [6]. Most cases are seen between June and November. Two peaks are generally seen, with the first peak occurring during June-July and a smaller peak during October-November. Simultaneous infections with *Babesia* and *B. burgdorferi* have been reported after a single tick bite, as the tick *I. scapularis* is a known vector for both of these organisms [7]. 

Patients are known to develop signs of illness within one to two weeks of the tick bite. The typical symptoms include fever, malaise, myalgia, and headache, and are seen in 25% to 50% of the patient population. The clinical manifestations can range from mild illness to severe disease, including septic shock. Patients with compromised immunity (old age, immunosuppressive drugs, malignancy, chronic illness) are more likely to develop severe disease [1].

Severe diseases with complications, including acute kidney injury, rhabdomyolysis, disseminated intravascular coagulopathy, and acute respiratory distress syndrome, require hospitalization and intensive care unit admission. Rhabdomyolysis and acute kidney injury are rarely encountered complications of anaplasmosis. On review of the available literature, two instances with similar presentations have been reported. Jeong Min Cho et al. describe a patient who presented with systemic signs of rickettsial disease and was found to have rhabdomyolysis and AKI on lab work, which resolved with 10 days of doxycycline [8]. Zainab Shahid et al. describe a case of a 72-year-old male who was suspected to have septic arthritis and was found to have rhabdomyolysis and AKI on workup. He later tested positive for Anaplasmosis and responded well to doxycycline [9]. The mechanism of rhabdomyolysis remains unknown. Increased activation of macrophages and increased release of cytokines are considered to be associated with anaplasmosis-induced rhabdomyolysis [10].

A presumptive diagnosis of Anaplasmosis can be made in patients with appropriate epidemiologic exposures and unexplained systemic febrile illness with no alternative explanation. Given the risk for rapid progression to severe systemic disease, the CDC recommends starting treatment whenever the infection is clinically suspected without waiting for definitive diagnostic test results. PCR, serology, and peripheral blood smear examination are all definitive tests. PCR-based testing has high sensitivity and specificity during the first week of an illness [11]. A peripheral blood smear can help identify morulae in the neutrophils, which are seen in 20% to 80% of patients [12]. Serological tests measure titers of specific immunoglobulin M (IgM) and immunoglobulin G (IgG) levels, and a definitive diagnosis requires at least a fourfold change in the IgG titer between the acute and convalescent stages, with at least one of the IgG titers being at least 1:64 to 1:80 [13].

The treatment of choice for both adult and pediatric populations is doxycycline. For pregnant patients, rifampin is an alternative treatment option [10]. The CDC recommends 100 mg of doxycycline twice daily for 10 to 14 days to cover the possibility of concurrent Lyme disease infection. Within the first 24-48 hours after starting antimicrobial therapy, there is a rapid clinical response [14].

## Conclusions

Anaplasmosis can manifest as a mild, self-limiting illness in healthy individuals or as a severe disease in patients with risk factors for diminished host defense mechanisms, like old age, an immunosuppressed state, human immunodeficiency virus infection, organ transplantation, and cancer. In rare cases, it can present with rhabdomyolysis and acute kidney injury. Our case highlights the importance of making a presumptive diagnosis of anaplasmosis based on constitutional symptoms and laboratory abnormalities like leukopenia, thrombocytopenia, and transaminitis in patients living in endemic areas and starting empiric treatment while awaiting confirmatory results, which can lead to rapid improvement and resolution of the condition. 
